# Chronic IL-21 exposure reshapes pulmonary environment, elevating risk of respiratory diseases

**DOI:** 10.1007/s00018-026-06106-3

**Published:** 2026-02-14

**Authors:** Sudhanshu Agrawal, Hugo Oyamada, Nicholas Steven Korvink, Siyi Zhou, Cleonice Alves de Melo Bento, Farah Rahmatpanah, Veedamali S Subramanian, Anshu Agrawal

**Affiliations:** 1 Division of Basic and Clinical Immunology, Department of Medicine, University of California Irvine, Irvine, CA 92697 USA; 2 Department of Microbiology and Parasitology, Federal University of the State of Rio de Janeiro, Rio de Janeiro, Brazil; 3 Department of Microbiology, Immunology and Parasitology, Rio de Janeiro State University, Rio de Janeiro, Brazil; 4 Department of Pathology, University of California Irvine, Irvine, CA 92697 USA; 5 Division of Gastroenterology and Hepatology, Department of Medicine, University of California Irvine, Irvine, CA 92697 USA

**Keywords:** Lung, Inflammation, IL-21, CD8 T cell dysfunction, Senescence, Infection

## Abstract

Age-related pulmonary diseases pose a significant health burden, yet the underlying mechanisms remain poorly understood. This study investigates the role of interleukin-21 (IL-21) in driving age-associated changes in lung function and immune responses. Using both murine models and human samples, we demonstrate that IL-21 induces a pro-inflammatory state in the lungs, characterized by increased levels of key inflammatory cytokines including TNF-α, IL-6, IL-33, CXCL-10, and IL-18. IL-21 exposure also promoted cellular senescence, evidenced by upregulation of senescence-associated genes and increased frequencies of KLRG1-positive T cells. Notably, IL-21 treatment led to significant alterations in lung macrophage phenotype and function. We observed increased lipid accumulation in macrophages, accompanied by upregulation of lipid uptake receptors TREM-2 and CD36. These changes were associated with elevated TGF-β secretion, suggesting a potential mechanism for IL-21-induced pulmonary fibrosis. Furthermore, IL-21 exposure resulted in impaired antiviral responses, characterized by reduced MHC-II expression on macrophages and diminished IFN-α production in response to viral challenges. Importantly, aged mice exhibited a lung phenotype strikingly similar to that induced by IL-21 treatment in young mice, including increased inflammation, cellular senescence, and altered macrophage lipid metabolism. Furthermore IL-21 expression was found to be elevated in the lungs of Idiopathic pulmonary fibrosis (IPF) patients compared to controls. These findings suggest that age-related elevation of IL-21 levels may be a key driver of pulmonary dysfunction in the elderly.

**Graphical Abstract:**

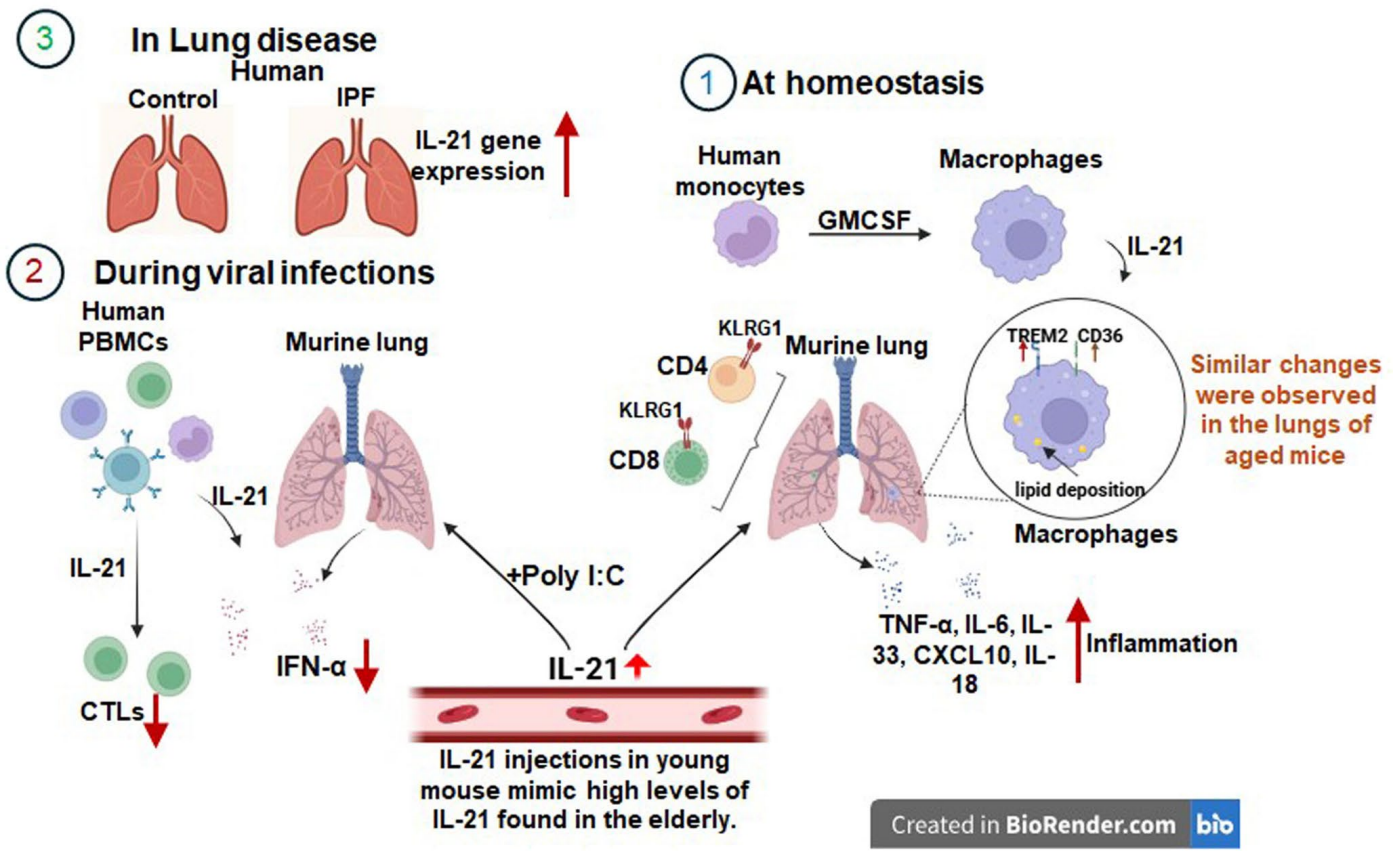

## Introduction

 As the global population ages, the proportion of individuals over 60 is expected to nearly double by 2050. Aging brings molecular and physiological changes that significantly affect lung health, resulting in altered lung function, reduced pulmonary remodeling, and diminished regenerative capacity [1]. With advancing age, lung capacity and resilience decline, resulting in an increased susceptibility to a wide range of pulmonary diseases [2]. Aging is a major risk factor not only for acute lung infections but also for chronic respiratory diseases such as chronic obstructive pulmonary disease (COPD), idiopathic pulmonary fibrosis (IPF), and pulmonary hypertension. The incidence and severity of lung infections escalate with age, often leading to worse clinical outcomes, prolonged hospitalizations, and life-threatening complications such as sepsis and acute respiratory distress syndrome (ARDS), as observed during the recent COVID-19 pandemic [3–6]. With the aging population growing, understanding how changes in pulmonary and immune cell interactions drive infection susceptibility and chronic disease is crucial for developing targeted therapies.

Increased lung inflammation, cellular senescence, and immune dysfunction are recognized as key contributors to age-related pulmonary decline. These interconnected processes lead to a cascade of detrimental changes, including impaired tissue repair, disrupted epithelial barrier integrity, persistent low-grade inflammation (“inflammaging”), reduced mucociliary clearance, and dysregulated immune cell recruitment and activation [7–9]. Chronic or repeated inflammatory insults compromise the epithelial barrier, increase vascular permeability, and promote excessive infiltration and activation of immune cells, resulting in tissue injury, extracellular matrix remodeling, and fibrosis [10–12]. We have previously shown that increased baseline activation of dendritic cells from older adults enhances the permeability of airway epithelial cells [13]. Inflammatory mediators such as IL-6, IL-1β, and TNF-α not only sustain inflammation but also drive the induction of cellular senescence. Senescent cells, which accumulate in epithelial, endothelial, and immune compartments of the lung, adopt a senescence-associated secretory phenotype (SASP) characterized by the release of pro-inflammatory cytokines, chemokines, proteases, and growth factors. This SASP exacerbates local tissue damage, promotes fibroblast activation, impairs epithelial regeneration, and creates a selfamplifying loop that perpetuates inflammation. Simultaneously, immune dysfunction compromises both innate and adaptive immunity: alveolar macrophages and dendritic cells exhibit increased baseline activation, yet show reduced responsiveness to infections, as previously reported [14–16]. These alterations contribute to a chronic, unresolved inflammatory environment that further reinforces senescence and immune dysregulation. Together, these processes culminate in diminished pulmonary function, increased susceptibility to infections, and the progression of age-related lung diseases such as COPD, IPF, and pneumonia. Despite increasing recognition of their roles, the upstream mechanisms and molecular drivers of inflammation, senescence, and immune dysfunction in the aging lung remain poorly understood.

Interleukin-21 (IL-21) belongs to the common gamma chain (γc) cytokine family and is produced by various immune cells, with activated CD4 + T cells, particularly T follicular helper (TFH) cells,and natural killer (NK) T cells serving as its primary sources [17–20]. IL-21 acts on B cells to enhance their differentiation to antibody secreting B plasma cells. In addition, IL-21 also enhances the cytotoxic function of CD8 T cells. Chronic viral infections also lead to the production of IL-21 where it preserves the functions of CD8 T cells and plays an essential role in containing the infection [21, 22]. IL-21 exerts its effects through binding to the IL-21 receptor (IL-21R), which activates downstream signaling via the JAK-STAT pathway [23]. The IL-21 receptor is broadly expressed on both immune and non-immune cells, including lung epithelial cells and fibroblasts [17, 24, 25]. A role for IL-21 in lung infections has been suggested by several studies. For example, IL-21 promotes the pathogenic inflammatory effect of Pneumonia Virus (PVM) of mice by enhancing neutrophil recruitment and acute respiratory distress [26]. The IL-21/IL-21R axis also aggravated chlamydial lung infection by inhibiting Th1 and Th17 responses [27]. Beyond its role in infections, IL-21 has been implicated in lung fibrosis, as IL-21R knockout mice exhibit resistance to fibrosis [28]. Furthermore, IL-21R expression is significantly upregulated in human fibrotic lung tissues, suggesting a direct link to disease pathologies [29]. Moreover, IL-21-driven M2 macrophages contribute to the pathogenesis of pulmonary arterial hypertension [30]. Collectively, these findings underscore the critical role of IL-21in various lung pathologies.

Despite existing research, the role of IL-21 in lung diseases remains relatively underexplored. Our previous findings indicate that IL-21 levels are elevated in older adults and correlate with CMV seropositivity [31, 32]. In this study, we examined how elevated IL-21 levels impact the pulmonary microenvironment and contribute to age-associated changes, including inflammation, susceptibility to infections, and cellular senescence.

## Materials and methods

### Blood

Peripheral blood samples were collected from healthy adult volunteers aged 22 to 52 years, with assistance from the Institute for Clinical and Translational Science (ICTS) at UC Irvine. The study protocol was approved by the Institutional Review Board (IRB) at the University of California, Irvine. All participants provided written informed consent prior to sample collection.

### IL-21 and influenza stimulation of peripheral blood nuclear cells (PBMCs)

PBMCs were separated from the blood by density gradient centrifugation using lymphocyte separation media (Mediatech). PBMCs were treated with IL-21 (10ng/ml) for 72 h. For influenza experiments- PBMCs were treated with IL-21 for 72 h, followed by stimulation with 10 µg/ml inactivated influenza virus A/PR/8/34 (Charles River) during the final 24 h. Subsequently, the cells were collected for flow cytometry and supernatants were stored for multiplex assays. PBMCs collected after stimulation for 24 h were stained with fixable viability stain 510 for live/dead cells exclusion as per manufactures instruction (BD Biosciences). The cells were then washed, and surface stained for CD4, CD8 T cells and monocytes using specific antibodies for 30 min at RT in dark. Subsequently, the cells were washed and fixed using 2% PFA. The required FMO and isotype controls were prepared the same way. Cells were acquired by BD FACS Celesta (Becton-Dickenson, San Jose, CA) equipped with BVR laser. Forward and side scatters and singlets were used to gate and exclude cellular debris. Thirty thousand cells were acquired per sample. Analysis was performed using FLOWJO software (Ashland, OR). Monocytes were identified by CD14^+^/HLA-DR^+^.

### Macrophage differentiation

Monocytes were purified from PBMCs by CD14 positive selection kit (Stemcell Sep, Vancouver). Purified monocytes were differentiated into macrophages by culturing them with GMCSF 50 ng/ml. After 6 days, macrophages were collected and used for stimulation.

### Mice and IL-21 injections

C57BL/6 mice (Jackson Laboratories) were used for these experiments. All animal procedures complied with NIH guidelines and were approved by the Institutional Animal Care and Use Committee at the University of California, Irvine. Mice were housed under standard laboratory conditions (temperature: 20 ± 1 °C; humidity: 70 ± 10%; 12-h light/dark cycle) with unrestricted access to regular rodent chow and water. At 3 months of age, healthy mice received intravenous injections of recombinant mouse IL-21 (50 µg/kg; BioLegend, San Diego, CA) twice per week for five total doses, following established protocols [13]. Control animals received phosphate-buffered saline (PBS). Forty-eight hours after the final injection, animals were euthanized. Broncho alveolar lavage (BAL) fluid and lungs were collected. BAL was centrifuged to collect cells which were stained for neutrophils and macrophages using flow cytometry. The supernatant was used for assay of cytokines/chemokines using multiplex. Lungs were used for assaying cellular and other changes.

### Polyinosinic: polycytidylic acid (Poly I:C) experiments

Mice (8 weeks) were treated with IL-21 as above. After the last dose, Poly I:C (Invivogen) 50 µg in PBS was instilled intranasally (IN) in 50 µl volume [33]. Control mice were given only PBS. One day later the mice were sacrificed, and BAL fluid was collected and assayed for various cytokines and infiltrated cells. Lungs were collected for other assays.

### Flow cytometry

For flow cytometry analysis, lungs of mice were digested in collagenase. The cells were then collected and stained with BD Horizon™ Fixable Viability Stain 510 for identification of live cells, as per manufacturer’s instructions. Cells were then washed, and surface stained for CD45 (clone 30-F11) PerCP, CD11b (cloneM1/70) BV605,CD11c (clone N418) APC, MHC-II (clone M5/114.15.2) Spark Red™ 718, MHC-I PE, Ly6G (clone 1A8)APC, CD36 (clone ZND36-6) FITC, CD4 (clone GK1.5) Brilliant Violet 605, CD8 (clone 53 − 6.7) Brilliant Violet 421, KLRG-1 (clone 2F1/KLRG-1) PE, (Biolegend, CA). After staining, cells were washed and fixed /permeabilizated with BD Cytofix/Cytoperm™ kit (Becton-Dickenson, San Jose, CA), following the manufacturer's instructions. After washing, cells were intracellular stained for CD68 (clone FA-11) APC, and TREM-2 (clone 6E9) PE, Granzyme B (clone QA16A02) APC, markers. Acquisition was done on BD FACS Celesta (Becton-Dickenson, San Jose, CA). Forward and side scatters were utilized to gate and eliminate cellular debris. Analysis was performed using FlowJo™ 10.10 software (BD Life Sciences, Ashland, OR).

For BODIPY staining cells prepared as above were incubated with BODIPY (boron-dipyrromethene) 493/503 dye for 15 min at 37 °C. Subsequently they were washed and stained for surface markers. Cells were then washed, acquired on a flow cytometer, and analyzed for lipid content in gated macrophages.

### Real time PCR of lung samples

cDNA was prepared from total RNA isolated from mouse lungs using established procedure (REF). RT-qPCR was performed as described before (REF) using mice specific primers for p16, p21, ACTα2, TNF-α, IL-6, CXCL-10 and β-actin (p16-F-5’-CCAACGCACCGAATAGTTACG-3’; R-5’- GCGCTGCCCATCATCATG-3’, p21-F-5’-GACACCACTGGAGGGTGACT-3’; R-5’- CAGGTCCACATGGTCTTCCT-3’, ACTα2 5’-F- GTCCCAGACATCAGGGAGTAA − 3’; R-5’- TCGGATACTTCAGCGTCAGGA-3’, TNF-α -5’-F- CATCTTCTCAAAATTCGAGTGACAA − 3’; R-5’- TGGGAGTAGACAAGGTACAACCC − 3’, IL-6-5’-F-GAGGATACCACTCCCAACAGACC-3’; R-5’-AAGTGCATCATCGTTGTTCATACA − 3’, CXCL-10-5’-F-CCAAGTGCTGCCGTCATTTTC-3’; R-5’-GGCTCGCAGGGATGATTTCAA-3’, β-actin-5’-F-ATCCTCTTCCTCCCTGGA-3’; R-5’-TTCATGGATGCCACAGGA-3’). These primers were synthesized by Integrated DNA technologies Inc (San Deigo, CA) or by Invitrogen (Carlsbad, CA). Relative p16, p21, ACTα2, TNF-α, IL-6 and CXCL-10 mRNA expression levels were determined by normalizing Ct values with respective β-actin [34, 35].

### Statistical analysis

For statistical analysis, GraphPad Prism 10.0 software was utilized. Paired and unpaired Student’s *t* test was used, when applicable. For comparisons involving three or more groups, statistical analyses were performed using one-way ANOVA followed by Tukey’s multiple-comparison post hoc test. Values of *p* < 0.05 were considered significant. All tests were two tailed with 95% confidence interval.

## Results

### IL-21 induced changes in the lungs at homeostasis

**Fig. 1:**
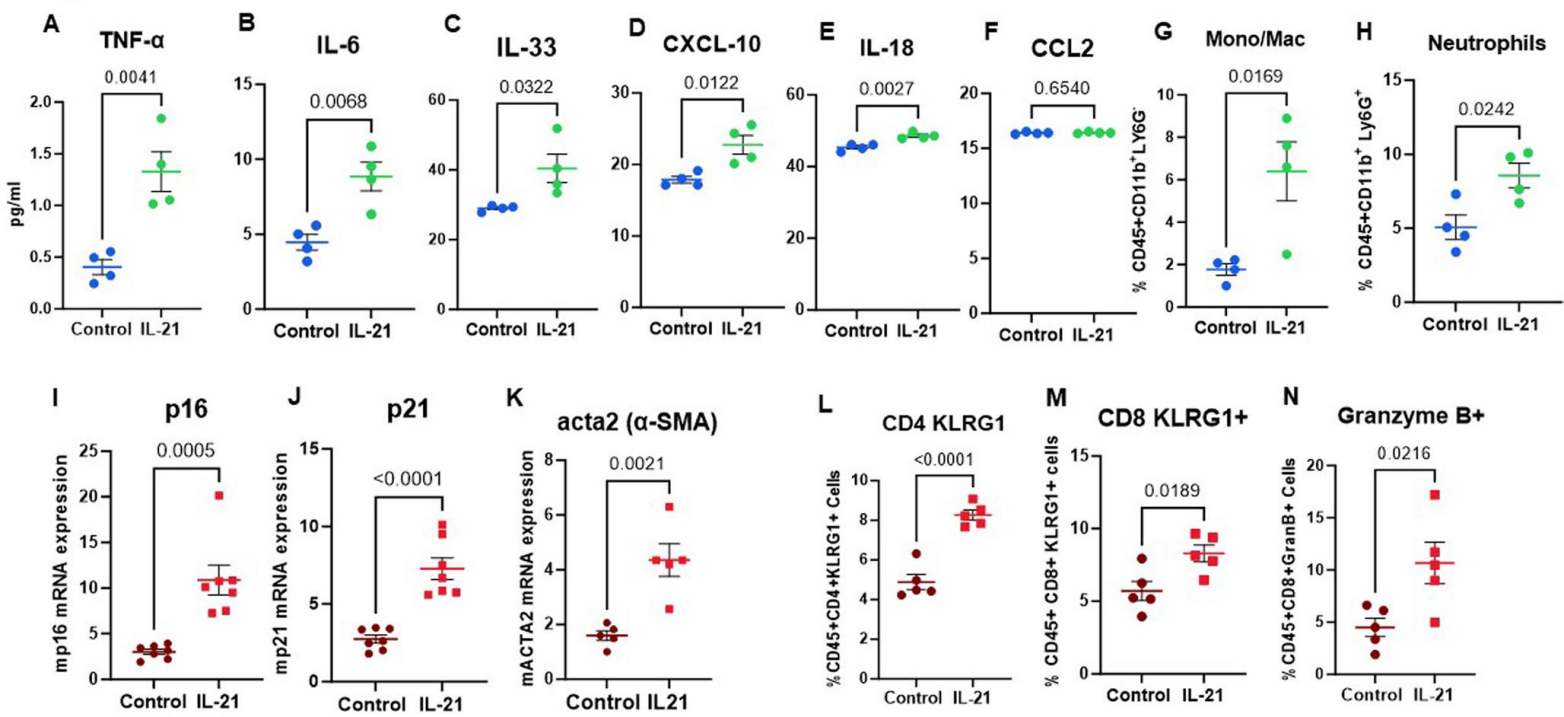
IL-21 induced changes in the lung at homeostasis. Mice were given 5 injections of IL-21. BAL and lungs were collected. The level of cytokines and chemokines were determined in the BAL fluid using multiplex. Dot blots depict the pg/ml levels of- **A**. TNF-α; **B**. IL-6; **C**. IL-33; **D**. CXCL-10; **E**. IL-18; **F**. CCL2. Dot blots depict the percentages of – **G**. Monocytes/macrophages; **H**. Neutrophils is the BAL fluid as determined by flow cytometry. Dot blots show relative mRNA expression levels of **I**. p16^INK4A^; **J**. p21; **K**. acta-2 in lung tissue, as measured by RT-qPCR. Dot blots show the percentages of – **L**. CD4^+^KLRG^+^; **M**. CD8^+^KLRG^+^; **N**. CD8^+^Granzyme B^+^ in the lungs. Data are presented as mean ± SEM

Persistent inflammation within the respiratory tract underlies the pathogenesis of numerous chronic pulmonary diseases associated with aging including COPD, asthma, and pulmonary fibrosis [36]. To investigate the effect of IL-21 on lung inflammation, mice. were given recombinant IL-21 for 3 weeks for a total of five injections to mimic chronic IL-21 conditions. 24 h after the last injection BAL fluid was collected and assayed for infiltrating cells and various cytokines. Lung collected were assayed for other markers. Remarkably, IL-21 treatment enhanced the production of pro-inflammatory mediators, TNF-α, IL-6, IL-33, CXCL-10 and IL-18 **(**Fig. 1A-E**)** in the BAL indicating that IL-21 exacerbates pulmonary inflammation at homeostasis. CCL2 levels displayed no significant change **(**Fig. 1F**)**. In addition, we also observed increased infiltration of neutrophils and inflammatory monocytes in the BAL as determined by flow cytometry **(**Fig. 1G-H**)**.

The senescence of aged lungs is considered a major cause of lung related diseases. Senescence associated secretory phenotype (SASP) also leads to chronic inflammation. To investigate if IL-21 influences senescence, we determined the expression of senescence related genes p16^INK4A^ (p16) and p21 (CDKN1A) in the lungs using qPCR. The expression of both these genes was significantly upregulated in the lungs of IL-21 injected mice **(**Fig. 1I and J**)**. Additionally, we also observed an increased expression of acta-2 (alpha smooth muscle actin) in the lungs on IL-21 exposure **(**Fig. 1K**).** Acta-2 is a key characteristic of myofibroblasts, which are cells that play a role in lung remodeling and fibrosis [37, 38]. It is associated with myofibroblast differentiation and epithelial to mesenchymal transition [39].

KLRG1 is considered a marker of terminal differentiation and senescence in T cells. It is highly expressed in T cells from senescent patients [40–43]. Reports from literature suggest that IL-21 can influence the differentiation of T cells and result in the upregulation of KLRG1 during acute infections [44, 45]. However, whether chronic IL-21 levels influence KLRG1 expression in the absence of infection is not known. Our results revealed significantly increased percentages of CD4 and CD8T cells expressing KLRG1 in the lungs of mice injected with IL-21 **(**Fig. 1L and M**)**. IL-21 also modulates the cytotoxic activity of CD8 T cells [46]. In keeping with this we observed increased proportions of CD8 T cells expressing Granzyme B **(**Fig. 1N**)** upon IL-21 treatment. Thus, IL-21 appears to induce terminal differentiation/senescence in T cells.

Together these data indicate that chronic IL-21 enhances inflammation and senescence in the lungs.

### IL-21 impairs response to viral infections

**Fig. 2:**
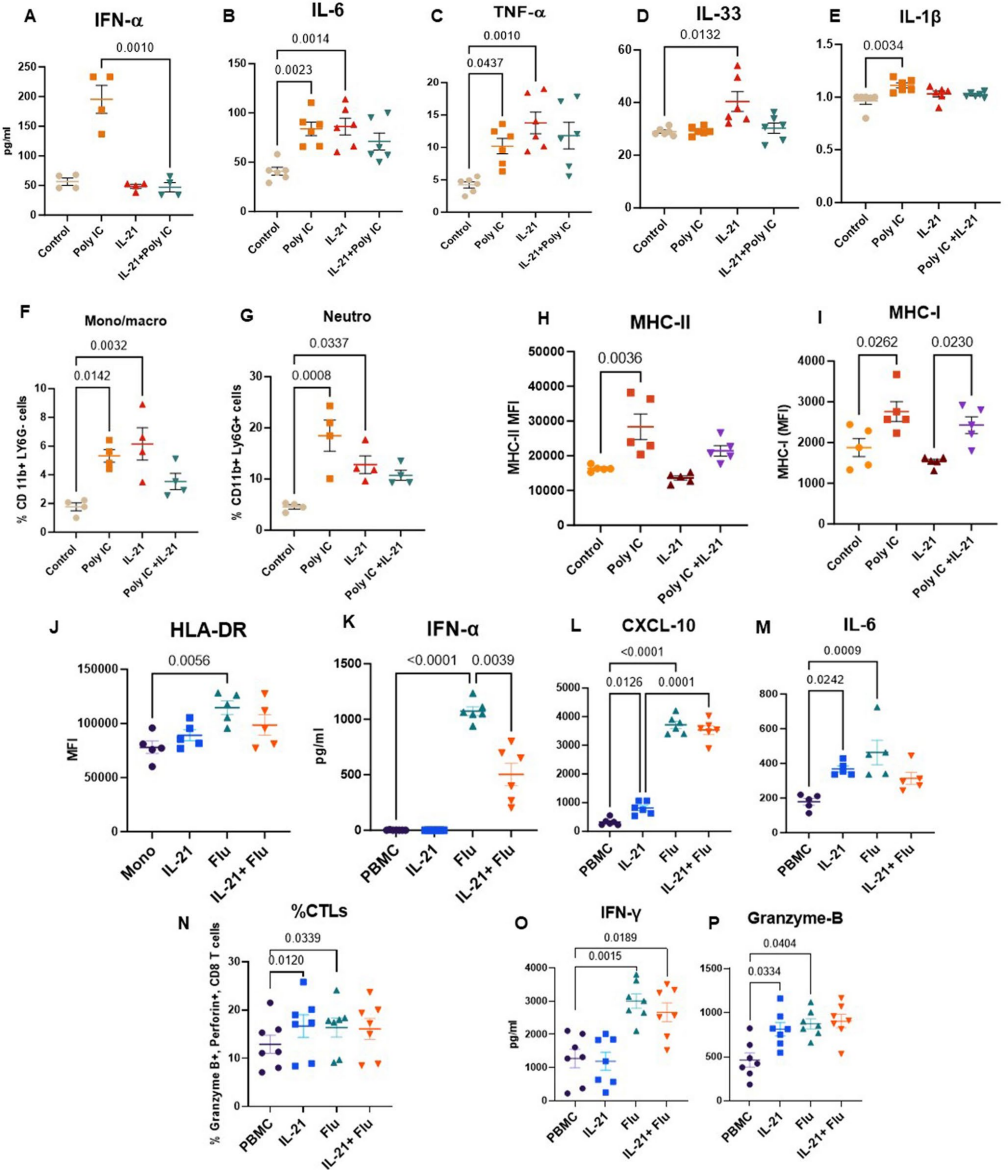
IL-21 impairs response to viral infections. Mice injected with IL21 were stimulated with Poly I:C in the last 24 h. Lungs and BAL were collected 24 h after the stimulation. Dot plots depict the concentration of cytokines and chemokines in the BAL. **A**. IFN-α; **B**. IL-6; **C**. TNF-α; **D**. IL-33; **E**. IL-1β; Dot plots show the percentages of **F**. monocytes; **G**. neutrophils in the BAL. Dot plots depict the MFI of **H**. MHC-II; **I**. MHC-I on gated lung macrophages. Human PBMCs were exposed to IL-21 and subsequently stimulated with influenza virus (flu). Dot plots show **J**. the MFI of HLA-DR on gated monocytes; Quantitation of mediators **K**. IFN-α, **L**. CXCL-10; **M**. IL-6 in the supernatants collected 24 h post infection. Dot plots depict- **N**. % of CTLs; **O**. IFN-γ; **P**. Granzyme B one week post influenza infection. Data are presented as mean ± SEM

Older adults display increased susceptibility to respiratory viral infections. To investigate the potential role of IL-21 on respiratory viral infections, IL-21 treated mice (as in Fig. 1) were exposed to Poly I:C, a synthetic analogue of viral double-stranded RNA in the last 24 h. Poly I:C is a ligand to TLR3 and is used experimentally to model viral infections in vivo [47, 48]. One day later the mice were sacrificed, and BAL fluid was collected and assayed for various cytokines. IL-21 pretreatment resulted in inhibition of IFN-α secretion **(**Fig. 2A**)** in response to Poly I:C. IL-6, TNF-α and IL-33 displayed increased production at baseline but were not increased by Poly I:C stimulation in the IL-21 treated group **(**Fig. 2B-D**).** IL-1β levels were elevated in the Poly I:C-treated group compared to controls; however, no such increase was observed in the IL-21 + Poly I:C group relative to either the control or IL-21-treated groups **(**Fig. 2E**).**

We also assessed monocyte and neutrophil infiltration in the BAL. As shown in Fig. 2F and G, Poly I:C stimulation significantly increased the infiltration of both monocytes and neutrophils. IL-21 alone enhanced baseline infiltration; however, no further increase was observed with Poly I:C stimulation in the presence of IL-21.

Poly I:C treatment is known to activate innate immune cells such as macrophages. To assess this activation, we analyzed the expression of MHC-II and MHC-I on macrophages from lungs. As expected, Poly I:C significantly upregulated MHC-II expression; however, this increase was attenuated in mice pretreated with IL-21 **(**Fig. 2H**).** MHC-I expression displayed no significant change (Fig. 2I).

Next, we determined whether IL-21 also attenuated the response to viral infections in humans. To examine the effect of IL-21 on the innate and adaptive responses of PBMCs to influenza, PBMCs were exposed to IL-21 for 72 h, followed by stimulation with 10µg/ml inactivated influenza virus A/PR/8/34 (Charles River) during the final 24 h. Influenza virus was used in place of Poly I:C to confirm that the blunted immune response is a consistent feature across different viral stimuli. Flow cytometry analysis demonstrated that while influenza stimulation significantly upregulated HLA-DR expression on gated monocytes, this upregulation was absent in the IL-21 exposed influenza stimulated group (Fig. 2J). Additionally, IL-21 exposure significantly decreased IFN-α secretion in response to influenza (Fig. 2K). CXCL-10 levels were significantly elevated in the influenza-stimulated groups, both with and without IL-21 treatment **(**Fig. 2L**)**. Notably, IL-21 alone also enhanced CXCL-10 production at baseline. As expected, influenza stimulation increased IL-6 production; however, this upregulation was not observed in the IL-21 treated group (Fig. 2M). Interestingly, IL-21 alone led to increased IL-6 levels at baseline.

We also assessed IL-21’s impact on adaptive immunity, focusing on CD8 T cells. PBMCs were stimulated with influenza with and without IL-21 supplementation. Cells and supernatants were collected on day 7 post-stimulation. IL-21 alone increased the percentage of granzyme B and perforin-expressing cytotoxic CD8 T cells (CTLs), but this effect was not further enhanced by influenza stimulation (Fig. 2N). Interestingly, IFN-γ levels increased significantly with influenza in both conditions **(**Fig. 2O**)**, while granzyme B levels in the supernatants mirrored the intracellular expression patterns (Fig. 2P).

In summary, IL-21 dampens the immune response to viral infections by impairing both innate and adaptive immune functions.

### IL-21 enhances lipid accumulation in pulmonary macrophages

**Fig. 3:**
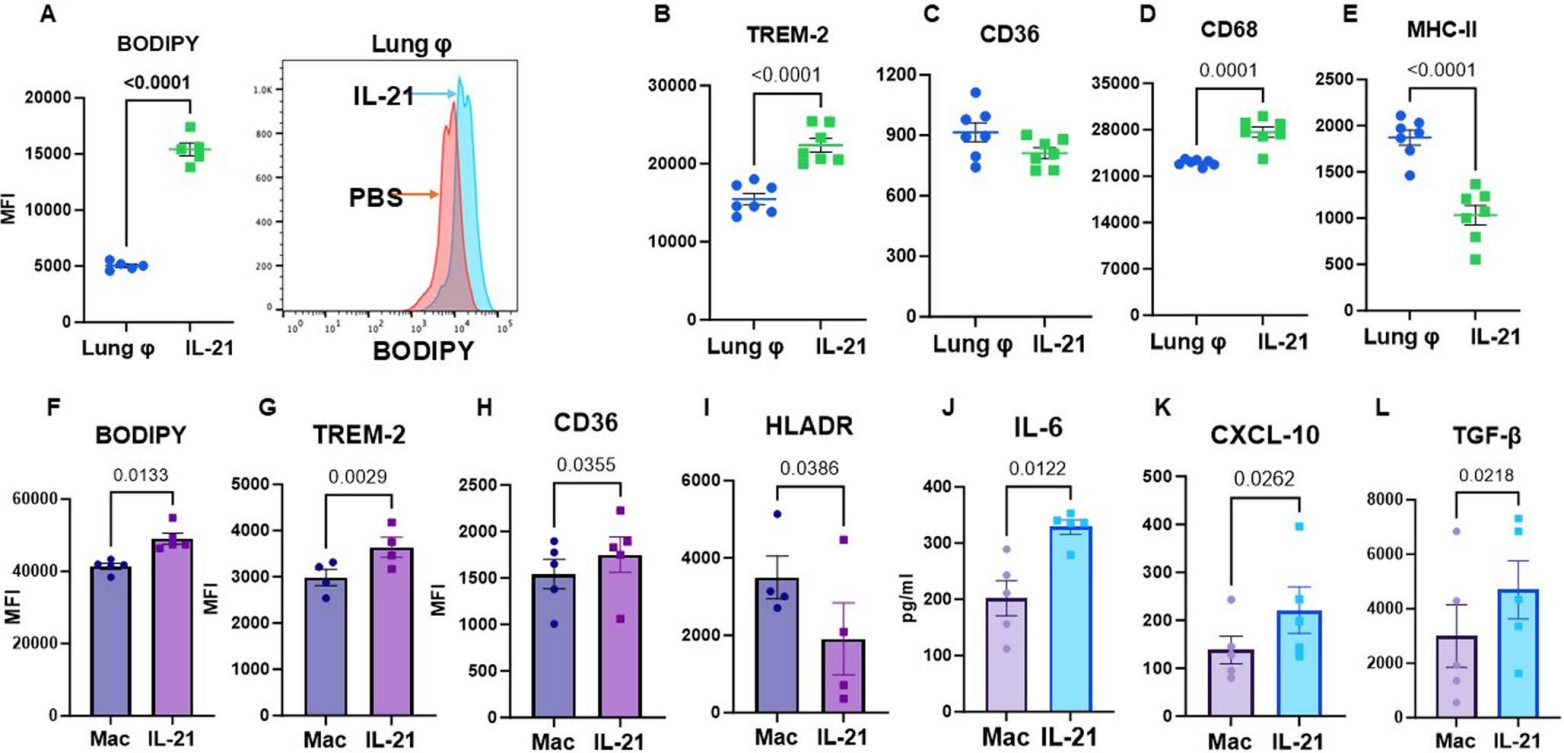
IL-21 enhances lipid accumulation in pulmonary macrophages. Mice were injected with IL-21 twice weekly for a total of 5 injections. The expression of various molecules and lipid accumulation in lung macrophages (lung φ) was determined by flow cytometry. Dot plots depict the mean fluorescence intensity (MFI) of- **A**. BODIPY; **B**. TREM-2; **C**. CD36; **D**. CD68; **E**. MHC-II on CD45^+^ CD11b^+^ gated lung φ. Data is mean ± SEM Human monocytes differentiated macrophages (Mac) were exposed to IL-21 for 72 h. Graphs show the MFI of **F**. BODIPY; **G**. TREM-2; **H**. CD36; **I**: HLA-DR on Mac. Supernatants were assayed for **J**. IL-6; **K**. CXCL-10; **L**. TGF-β using specific ELISAs. Data are presented as mean ± SEM

We have previously demonstrated that IL-21 induces lipid accumulation and other significant changes in microglia [49]. Given the critical role of pulmonary macrophages in both host defense and tissue repair, we next examined how IL-21 affects these cells. CD45⁺ CD11b⁺ cells from the lungs were analyzed for lipid accumulation using BODIPY staining. Lung macrophages (lung φ) from IL-21 injected mice displayed significantly increased lipid uptake compared to controls **(**Fig. 3A**)**. To investigate the underlying mechanism, we assessed the expression of lipid-sensing and uptake receptors TREM-2 and CD36, both implicated in lung injury and fibrosis [40–43]. IL-21 markedly upregulated TREM-2 expression on lung φ (Fig. 3B**)**, while CD36 expression remained unchanged **(**Fig. 3C**)**. Additionally, IL-21-treated macrophages exhibited reduced MHC-II expression and increased CD68 expression (Fig. 3D and E), suggesting that although the cells are activated, their antigen-presenting capacity may be compromised.

We next examined whether IL-21 induces similar changes in human monocyte-derived macrophages (Macs) as those observed in murine AMs. To generate macrophages, monocytes were differentiated in the presence of GM-CSF, a method known to produce cells resembling alveolar Macrophages [50, 51]. Macs were treated with 10 ng/ml IL-21 for 72 h, then collected and analyzed. IL-21 treated Macs exhibited changes similar to those observed in lung macrophages from IL-21 injected mice. Specifically, there was a significant increase in BODIPY MFI, indicating lipid accumulation **(**Fig. 3F**)**. Expression of the lipid-sensing receptors TREM-2 and CD36 were also elevated (Fig. 3G and H). Further, IL-21 exposure reduced the expression of HLADR **(**Fig. 3I**)**. Cytokine/chemokine analysis of the supernatant revealed elevated secretion of IL-6 and CXCL-10, mirroring the cytokine profile observed in IL-21-treated lungs (Fig. 3J and K). Notably, TGF-β, a key mediator of fibrosis, was significantly increased in the supernatant following IL-21 exposure (Fig. 3L). These findings suggest that IL-21 induces comparable functional and phenotypic changes in human and murine macrophages, further supporting its role in modulating macrophage activity.

### Lungs of older mice mimic the changes observed in young mice after chronic IL-21 injections

**Fig. 4:**
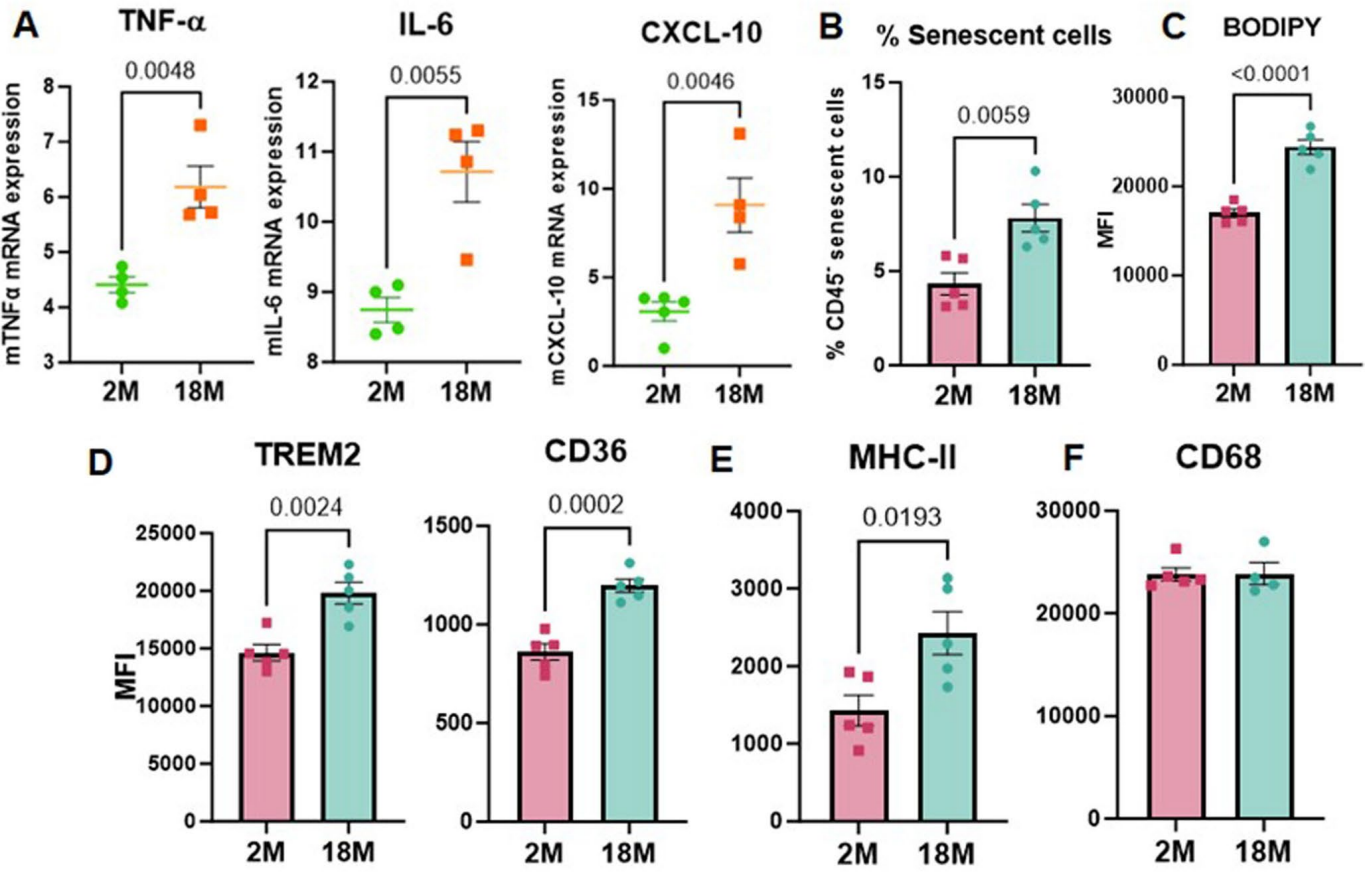
Lungs of older mice mimic the changes observed in young mice after chronic IL-21 injections. Graphs depict the changes in the lungs of 18M old mice as compared to 2M old mice. **A**. The level of expression of mTNF-α, mIL-6 and mCXCL-10 mRNA in the lungs; **B**. % of senescent cells in the lungs; The MFI of **C**. BODIPY; **D**. TREM-2, CD36; **E**. MHC-II; **F**. CD68 in gated lung macrophages. Data are presented as mean ± SEM.

Figures 1, 2 and 3 highlight significant lung alterations induced by chronic IL-21 administration. Given that IL-21 levels increase with age [31, 32], we investigated whether lungs from aged mice exhibited similar changes. We compared baseline inflammation, senescence, lipid accumulation, and other lung macrophage changes between 2-month-old and 18-month-old mice. Aged mice displayed increased baseline inflammation, evidenced by elevated gene expression of TNF-α, IL-6, and CXCL-10 in their lungs compared to young mice **(**Fig. 4A**)**, paralleling the inflammatory mediators upregulated by IL-21 injection. Since IL-21 also promoted senescence, we assessed senescent cells in aged and young lungs using the CellEvent Senescence Green Flow Cytometry Assay Kit (ThermoFisher). This reagent specifically detects senescent cells through β-gal activity and is compatible with multiplex analysis [52]. Aged mice showed a significant increase in senescent cells compared to young mice **(**Fig. 4B**)**. To evaluate lipid accumulation, lung macrophages from young and aged mice were stained with BODIPY dye, as done for IL-21 studies. Lung macrophages from aged mice exhibited markedly increased lipid accumulation **(**Fig. 4C**)**, mirroring IL-21-induced effects. Additionally, lipid-sensing molecules TREM-2 and CD36 were significantly elevated in the AMs of aged mice **(**Fig. 4D**)**. MHC-II expression, which regulates antigen presentation, was also increased in aged AMs, consistent with prior studies **(**Fig. 4E**)**. However, CD68 expression remained unchanged **(**Fig. 4F**)**.

Together, these findings indicate that aged lungs recapitulate several IL-21-induced changes, suggesting that elevated IL-21 levels during aging may drive these alterations.

### IL-21 gene expression is elevated in lungs of Idiopathic pulmonary fibrosis (IPF) subjects, and high IL-21 in controls mirrors IL-21 induced changes in murine lungs

**Fig. 5:**
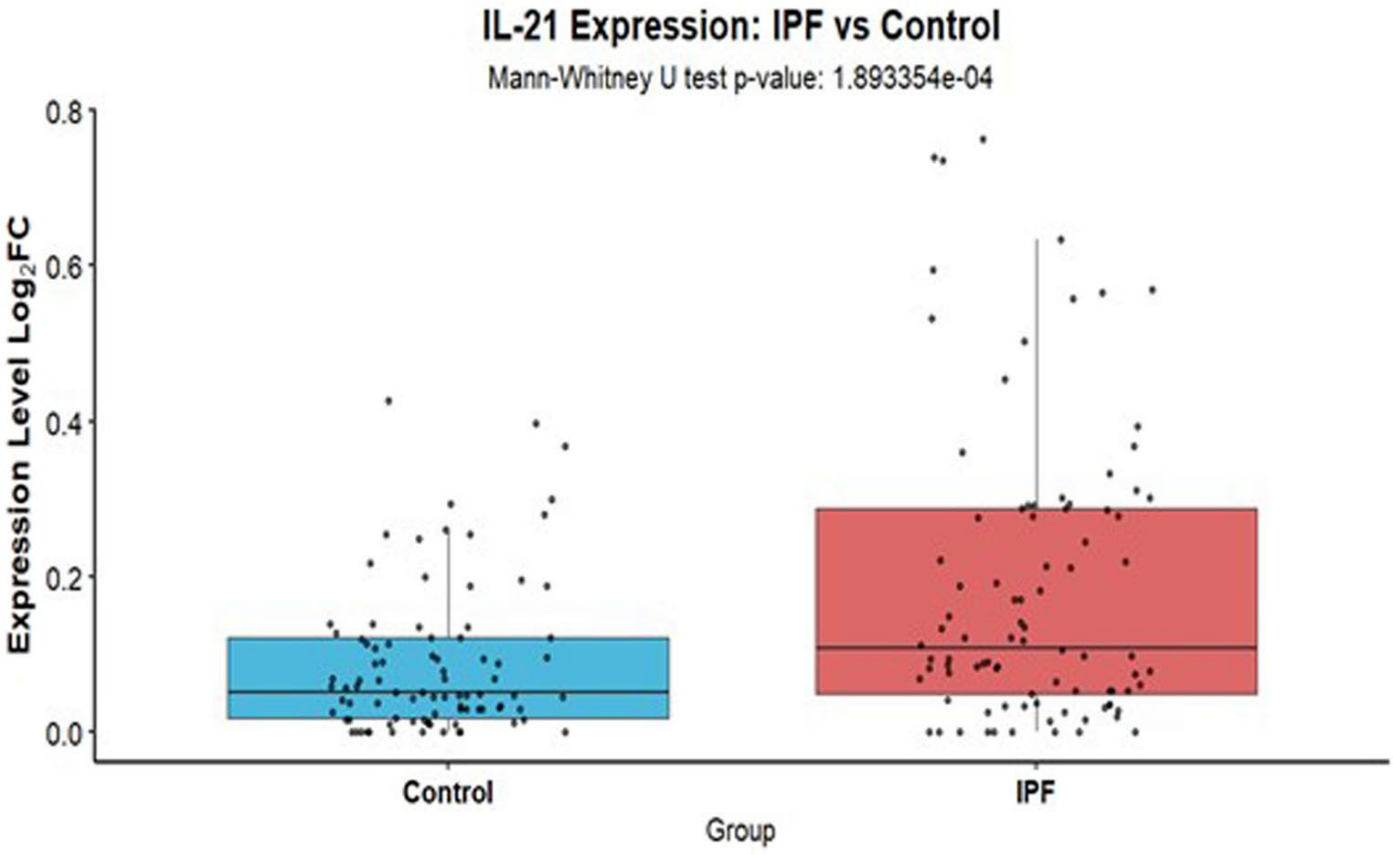
IL-21 gene expression is elevated in lungs of Idiopathic pulmonary fibrosis (IPF) subjects. Publicly available GSE 150910 was analyzed. Graph depicts the expression of IL-21 in the lungs of IPF and control subjects

To further validate the role of IL-21 in pulmonary diseases affecting the elderly, we leveraged publicly available sequencing data. Specifically, we analyzed raw gene expression counts from the Gene Expression Omnibus (GEO) dataset GSE150910 [53]. The dataset comprised of 103 control subjects and 103 IPF patients. IPF predominantly affects older adults, with aging-related impairments in tissue repair, immune regulation, and cellular homeostasis contributing to its onset and progression [54]. Raw counts were normalized using the Trimmed Mean of M-values (TMM) method from the R package edgeR to correct for differences in sequencing library size and composition between samples [55]. TMM scaling factors were calculated and applied to generate log2 counts-per-million (log-CPM) values, which were used for all subsequent statistical analyses. Mann-Whitney U statistical analysis revealed significant differences in IL-21 expression between IPF and control subjects **(**Fig. 5**)**.

Next, to investigate the impact of IL-21 expression levels on gene expression patterns of 18 genes related to lipid accumulation, immune dysfunction and senescence (chosen based on the results from Figs. 1 and 3), we stratified the control samples into ‘High IL-21’ and ‘Low IL-21’ groups based on their IL-21 transcript abundance. Our analysis focused exclusively on control samples, as these represent a condition analogous to the normal mouse lungs treated with IL-21 in our experiments in Fig. 1. This approach allows us to isolate the specific effects of elevated IL-21 levels on gene expression in healthy lung tissue, providing valuable insights into IL-21’s potential role in initiating pulmonary pathologies without the confounding factors present in diseased tissue.

The stratification threshold was established using the overall mean IL-21 expression calculated from the entire cohort, combining both control and IPF samples. This central cutoff approach provided a uniform benchmark that enabled direct comparison of the proportion of high and low expressing individuals between control (*n* = 103) and IPF (*n* = 103) populations. As shown in Table 1, control subjects with high IL-21 expression displayed significant changes in lipid uptake receptors, ABCA1 (ATP-binding cassette transporter A1), CD36, TREM-2, and PLIN2 (Perilipin-2) in keeping with our results of Fig. 3. Concurrently, we observed significant increases in the expression of CD8, Granzyme B and KLRG1 along with CXCL-10 mirroring our observations in mice and humans on IL-21 exposure. Additionally, the B plasma cell marker CD38 also showed significant upregulation [56]. Notably, we also detected increased expression of IRF4, a key transcriptional regulator of IL-21 production [57]. In contrast to these senescence genes displayed no significant changes between the high and low control group.

**Table 1:** Comparison of genes between control subjects exhibiting high and low IL-21 expression levels

Gene	Control_low_IL21	Control_high_IL21	Foldchange	*P* value	Significance
ABCA1	14.91812	19.31446	21.05860095	4.03491E-05	YES
CD28	1.284677	3.105513	3.532859285	2.18686E-14	YES
CD36	6.202255	5.111699	0.469580322	0.031126787	YES
CD38	4.439797	7.002532	5.908267477	7.17911E-07	YES
CD68	73.59401	83.56053	1000.505766	0.119866859	NO
CD8A	3.306234	6.565418	9.574410677	9.77741E-09	YES
CD8B	0.657017	1.267133	1.526381379	2.33296E-07	YES
CDKN1A	29.05721	26.21954	0.139885922	0.258144529	NO
CDKN2A	0.224532	0.223465	0.999260367	0.95622091	NO
CXCL10	8.561694	38.96939	1,424,391,215	2.36618E-08	YES
GZMB	0.945753	1.719227	1.709381382	4.47241E-07	YES
HIF1A	13.16403	13.49727	1.259840325	0.771813873	NO
IRF4	1.865916	4.02234	4.458084286	1.42684E-08	YES
KLRG1	0.368214	0.626715	1.1962357	1.81707E-09	YES
PLIN2	14.76224	11.54362	0.107424066	0.000594519	YES
SDC1	37.23829	35.47232	0.294029924	0.47581005	NO
TP53	1.234618	1.246136	1.008015762	0.842110164	NO
TREM2	7.698189	9.892201	4.575761974	0.015802661	YES

In conclusion, our analysis of public sequencing data demonstrates that IL-21 levels are increased in the lungs of IPF, a disease strongly associated with aging. More importantly, analysis of healthy controls reveals that elevated IL-21 expression correlates with significant changes in genes involved in lipid metabolism and immune activation, and cellular senescence corroborating our experimental results in mouse models.

## Discussion

This study elucidates the role of IL-21 in age-related pulmonary changes, focusing on senescence, infection susceptibility, and immune dysfunction. Our findings reveal that IL-21 exacerbates lung inflammation at homeostasis, a condition underlying various age-associated chronic pulmonary diseases including COPD, asthma, and pulmonary fibrosis [36]. Key inflammatory cytokines, including TNF-α, IL-6, IL-33, CXCL-10, and IL-18, were upregulated in the BAL, each contributing to specific aspects of lung pathology. CXCL-10 displayed increased expression in control lungs with high IL-21 transcripts. TNF-α and IL-6 are two key inflammatory cytokines linked to chronic inflammatory diseases and cancer [58, 59]. TNF-α is thought to be important for the development of pulmonary emphysema and heightening the fibrotic process [60]. IL-33 also plays a critical role in lung injury [61]. Inhibition of IL-33 via administration of neutralizing IL-33 antibody or IL-33 decoy receptor attenuates lung inflammation in murine models of asthma, COPD, and lung injury [62]. CXCL-10 and its receptor, CXCR3 signaling appears to be a critical factor for the exacerbation of the pathology of ARDS [63, 64]. Mice deficient in CXCL-10 or its receptor CXCR3 have decreased lung injury severity and increased survival in response to influenza virus infection [65]. Elevated endogenous IL-18 levels have been shown to promote inflammatory responses in the lung while simultaneously hindering effective bacterial clearance [66].

Moreover, IL-21 induced the expression of senescence-associated genes in the lung, potentially driving the senescence-associated secretory phenotype (SASP) and further perpetuating chronic inflammation. This suggests a dual mechanism by which IL-21 contributes to inflammation in the elderly: direct inflammatory stimulation and promotion of cellular senescence. We observed increased frequencies of KLRG1^+^ CD4 and CD8 T cells in IL-21 treated lungs. An increase in KLRG1 expression was also observed in human lungs with high IL-21 transcripts compared to those with low IL-21 transcripts. KLRG1, a marker of terminal differentiation and senescence in T cells, is associated with reduced functionality and impaired proliferative capacity [40–43]. KLRG1^+^ CD8 TEMRA cells have been reported to be increased in the lungs of patients with mild-moderate COPD [67]. Similarly, an increased proportion of peripheral KLRG1⁺ CD8⁺ T cells has been observed in older individuals with non-small cell lung cancer (NSCLC) which correlated positively with tumor size and TNM stage [68]. The increased expression of KLRG1 in our study therefore further supports IL-21’s role in promoting cellular senescence and immune dysfunction in the aging lung.

IL-21 treatment increased baseline CTL frequencies in both mice and human lungs, consistent with our prior findings showing elevated granzyme B expressing CTLs at homeostasis in older adults, which impairs antiviral responses [16]. Accordingly, IL-21 exposed mice exhibited a blunted response to the viral mimetic poly I:C, characterized by reduced inflammation and decreased macrophage MHC-II expression. Similar defects were observed in human PBMCs pretreated with IL-21 and challenged with influenza virus, including diminished IFN-α secretion and reduced CTL responses. By expanding the pool of nonspecific CTLs in the absence of infection, IL-21 appears to limit antigen-specific CTL responses upon viral challenge, as baseline CTL levels are already elevated. Together, these findings suggest that IL-21 skews immune homeostasis toward a state that is less capable of mounting effective antiviral responses, potentially contributing to age-related immune dysfunction during respiratory infections.

Other major changes induced by IL-21 included increased lipid accumulation in lung macrophages and human monocyte differentiated macrophages along with upregulation of lipid uptake receptors. Although the lung is intrinsically lipid-rich owing to surfactant phospholipids, macrophages normally do not become lipid-laden under homeostatic conditions [69]. However, the accumulation of lipid-laden macrophages in the lungs is increasingly recognized as a significant factor in lung pathophysiology, particularly during injury and inflammation where it may impair surfactant turnover, delay resolution of injury, and promote chronic tissue damage [70]. Lipid-laden macrophages often rely on fatty acid oxidation (FAO) and exhibit an anti-inflammatory phenotype, which may extend their lifespan and enable persistent, dysregulated signaling [71]. Such persistent macrophages are thought to promote fibrosis rather than resolving injury, exacerbating the transition from acute injury to chronic lung disease [72–75]. IL-21 also induced upregulation of TREM-2 which is reported to be involved in pulmonary injury and fibrosis [75–78]. TREM-2 critically regulates pulmonary macrophage lipid handling, influences sphingolipid metabolism, and modulates chemokine secretion, thereby contributing to fibrosis [76, 79, 80]. IL-21 also uniquely upregulated CD36 on human macrophages, enhancing uptake of oxidized phospholipids and potentially contributing to pulmonary fibrosis [77, 81]. Remarkably both CD36 and TREM2 were upregulated in human lungs with high IL-21. ABCA1 and PLIN2, both involved in lipid uptake and accumulation, were also upregulated, with ABCA1 playing a key role in lipid homeostasis and cholesterol efflux [82, 83]. PLIN2 coats lipid droplets, sequestering neutral lipids and protecting them from cytosolic lipases like ATGL, thereby preventing droplet coalescence and uncontrolled lipid breakdown, which promotes lipid accumulation [84, 85].

Further IL-21 exposure resulted in reducing the expression of antigen presenting molecules, MHC-II on macrophages, which may be a key factor contributing to the observed diminished response to infections. MHC class II plays a compensatory and protective role during respiratory viral infections by enhancing CD4⁺ T cell mediated immune responses. However, IL-21 exposure decreased MHC-II expression on macrophages thus compromising response to viral infection. Notably, secretion of TGF-β, a key mediator of fibrosis, was also significantly increased on IL-21 exposure. These data further suggest that IL-21 exposed macrophages are unlikely to contribute to the induction of KLRG1⁺ T cells, as the observed downregulation of MHC-II and TGF-β is not conducive to promoting terminal T-cell differentiation.

We have previously reported that circulating IL-21 levels increase with age and are associated with cytomegalovirus (CMV) seropositivity [31, 32]. Our current findings in aged mice demonstrate a baseline inflammatory profile that closely parallels the inflammatory mediators upregulated by IL-21 administration, suggesting a potential mechanistic link between age-related IL-21 elevation and chronic inflammation. They also show increased senescent cells and lipid-laden lung macrophages, reflecting IL-21 driven effects. In contrast to IL-21, we observed increased MHC-II expression on aged lung macrophages which is consistent with other studies [86, 87]. This suggests molecules other than IL-21 may be regulating MHC-II expression during aging. Together, these findings indicate that aged lungs recapitulate several IL-21 induced changes, suggesting that elevated IL-21 levels during aging may drive these alterations.

In conclusion, our study provides compelling evidence for the pivotal role of IL-21 in driving age-related pulmonary changes. Our findings reveal that IL-21 exacerbates lung inflammation, promotes cellular senescence, impairs antiviral responses, and alters macrophage lipid metabolism. These IL-21 induced changes closely mirror the alterations observed in aged lungs, suggesting that elevated IL-21 levels during aging may be a key driver of age-associated pulmonary dysfunction. The multifaceted effects of IL-21 on lung homeostasis and immune function offer new insights into the complex pathophysiology of age-related lung diseases. By promoting inflammation, cellular senescence, and lipid accumulation in macrophages, IL-21 creates an environment conducive to the development of chronic pulmonary conditions such as COPD, asthma, and pulmonary fibrosis. Furthermore, the IL-21 induced impairment of antiviral responses may explain the increased susceptibility to respiratory infections observed in elderly populations.

## Data Availability

Data will be made on reasonable request.
